# Supplementary material for: Efficient agricultural drip irrigation inspired by fig leaf morphology
Source: Nat Commun. 2023 Sep 23;14:5934. doi: 10.1038/s41467-023-41673-0 (PMC10518012; doi:10.1038/s41467-023-41673-0)
Supplement: Supplementary file 1 — Supplementary information [file 41467_2023_41673_MOESM1_ESM.pdf]

Supplementary Information

Efficient agricultural drip irrigation inspired by fig leaf morphology

Shijie Liu[1,2,3], Chengqi Zhang[1,3], Tao Shen[1], Zidong Zhan[1,3], Jia Peng[1,3], Cunlong Yu[1,3], Lei Jiang[1,2,3], Zhichao Dong[1,3]*

Affiliations

1 CAS Key Laboratory of Bio-inspired Materials and Interfacial Sciences, Technical Institute of Physics and Chemistry, Chinese Academy of Sciences, Beijing 100190, China

2 Suzhou Institute for Advanced Research, University of Science and Technology of China, Suzhou, Jiangsu 215123, China

3 School of Future Technology, University of Chinese Academy of Sciences, Beijing 100049, China

This PDF file includes:

Supplementary Figures 1 to 26
Supplementary Table 1

Other Supplementary Material for this manuscript includes the following:

Supplementary Movies 1 to 8

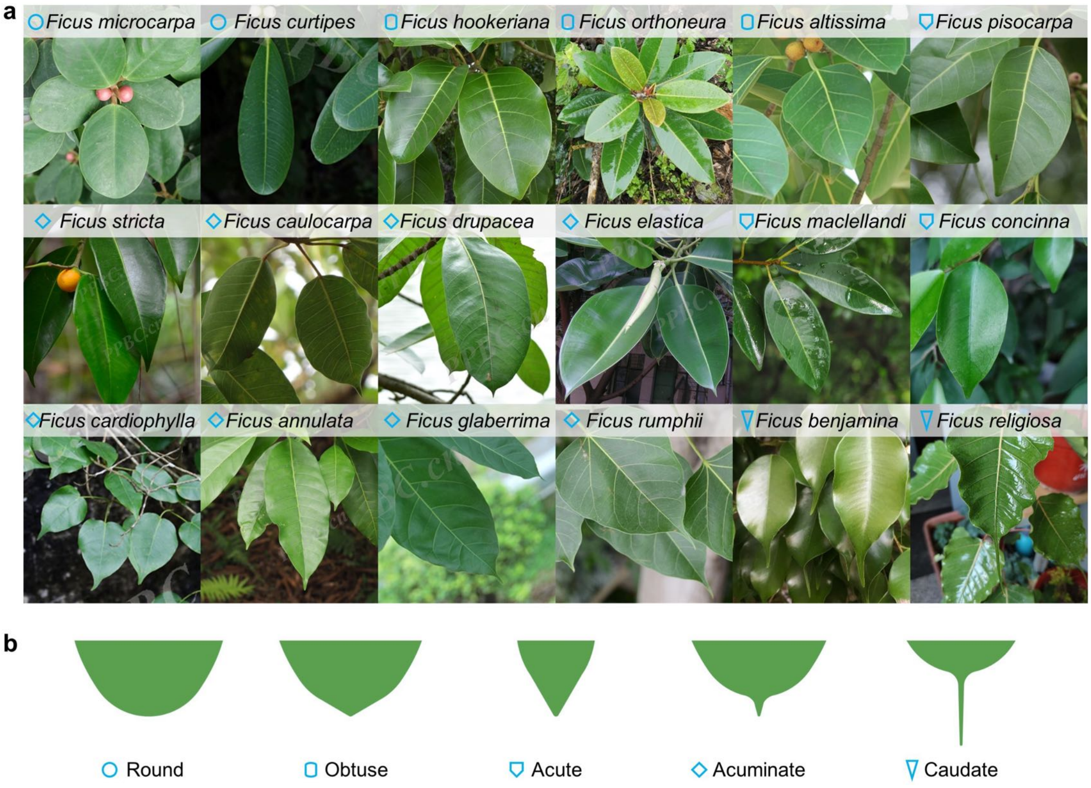

Supplementary Figure 1 | Leaf shape in the *Ficus* genus. a Photos of the investigated 18 leaf profiles in the *Ficus* genus. All these photos were extracted from http://ppbc.iplant.cn/, except the photograph of *Ficus religiosa*, which was taken from young bodhi trees planted in Beijing, China. **b** Leaf morphologies in the *Ficus* genus are classified into five groups based on apex shape.

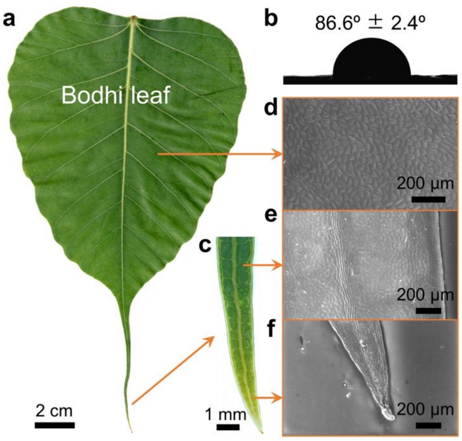

Supplementary Figure 2 | Wettability and morphology characterization of natural bodhi leaf. a Optical image of a natural bodhi leaf. **b** Water contact angle on the adaxial surface of natural bodhi leaf. **c** Light microscopy of natural bodhi leaf apex. The scanning electron microscopy of natural bodhi leaf at adaxial surface (**d**), leaf margin (**e**), and apex tip (**f**).

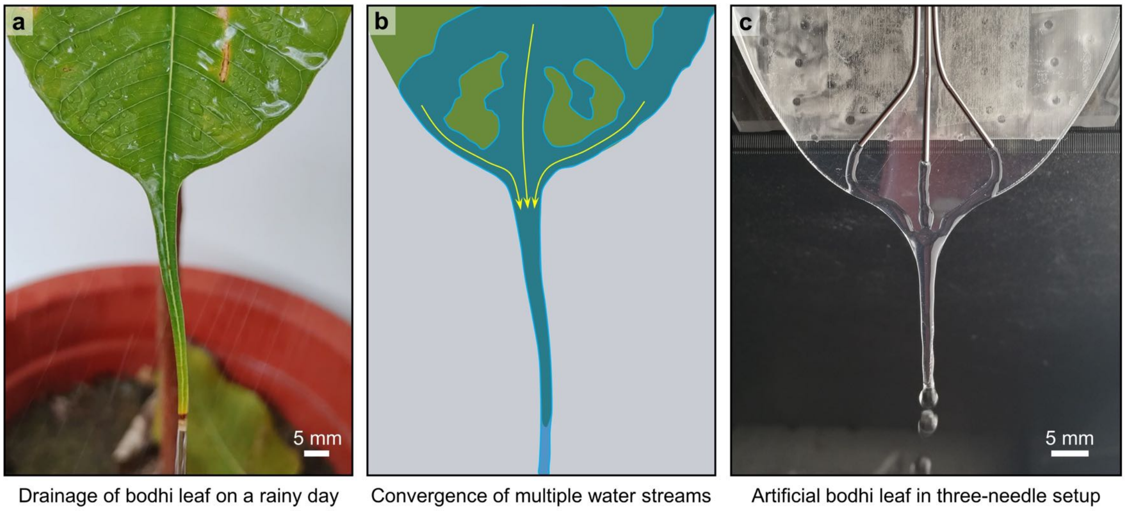

Supplementary Figure 3 | Convergence dynamics of multiple rainwater streams on a bodhi leaf. a Water drainage on a bodhi leaf in a rainy environment. **b** Sketch of the water flow on the bodhi leaf shown in (**a**). **c** Three-needle experimental setup for the evaluation of reverse curvature r/R.

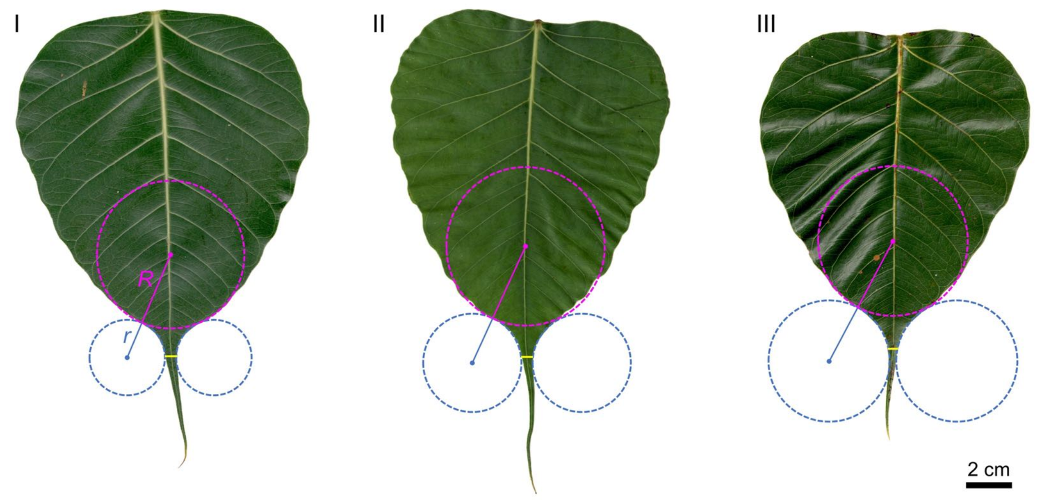

Leaf Shape	I	II	III
r/R	0.51	0.62	0.81
L_{apex}/W_{base}	9.8	12.3	8.3

Supplementary Figure 4 | Demonstration of geometrical measurement of bodhi Leaves. The convex curvature of the leaf body part is fitted by a purple circle and defined as $1/R$. The concave curvature of the leaf base part is fitted by a blue circle and defined as $1/r$. The reverse curvature is defined as r/R. The space between the two blue circles at the base of the leaf tip (short yellow line) is defined as the base width, W_{base}.

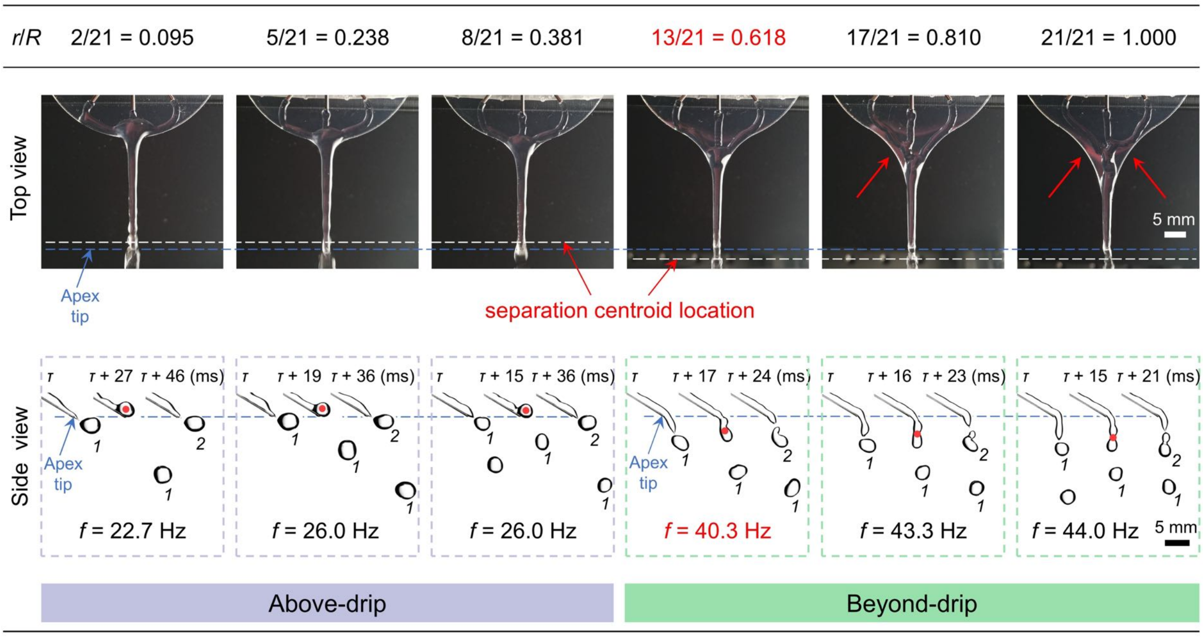

Supplementary Figure 5 | Top and side views of water flow dynamics on PET-based artificial bodhi leaf. The reverse curvature r/R ranges from 2/21 to 21/21. The injection flow rate Q is 32.0 mL min^{-1}, and inclination angle β is 30°. The red arrows in the top view indicate water flow breaking away from the leaf margin. The red dots in the side view indicate the separation centroid of the newly forming drop.

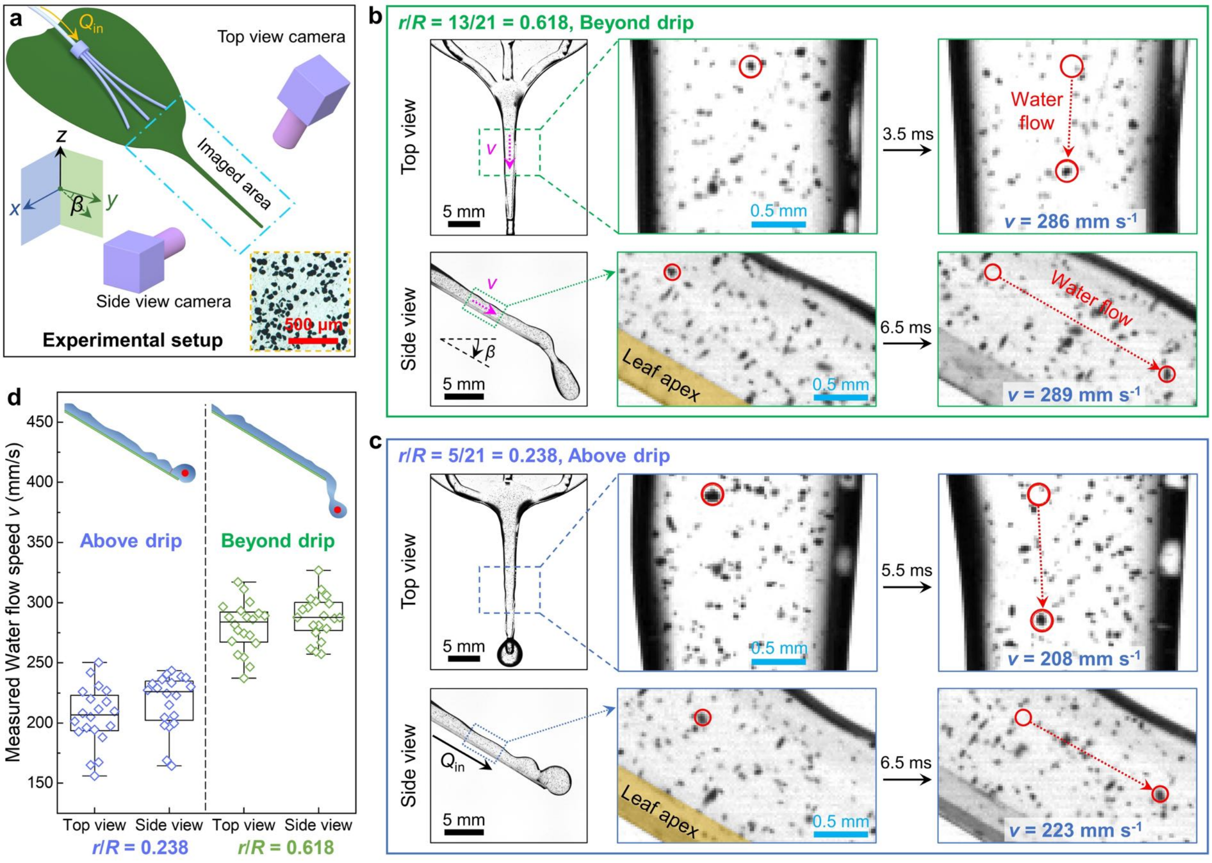

Supplementary Figure 6 | Effects of reverse curvature on water flow speed on artificial bodhi leaf apex. a Scheme of the experimental setup for measuring water flow speed *v* on artificial bodhi leaf apex. Water flow dynamics were recorded and analyzed from both top and side views. Inset: microscopy image of nylon particles with an average size of 50 μm, which were added into water to reflect the water flow speed *v* on the leaf apex. The scale bar in **a** is 500 μm. **b** Water flow dynamics on bodhi leaf apex with reverse curvature *r*/*R* of 0.618 in top view and side view. Q_{in} is 32 mL min^{-1} and β of 30°. The green dashed boxes mark the analyzed area. The movement of nylon particles along the water flow direction was tracked, and the average moving speed was calculated. **c** Water flow dynamics on bodhi leaf apex with reverse curvature *r*/*R* of 0.238 in top view and side view. **d** Measured *v* of bodhi leaf apex with *r*/*R* of 0.238 and 0.618. For each sample, the measured *v* in the top view was comparable to that in the side view. Under the same flow condition, the measured water flow speed *v* on *r*/*R* of 0.618 was significantly higher than that on *r*/*R* of 0.238. The convergence dynamics on *r*/*R* of 0.618 reduced the kinetic energy loss compared to that of *r*/*R* < 0.618. For the box plots in **d**, the bounds and centre line of boxes show the 25/75 percentiles and median values, and the upper and lower whiskers show maxima and minima values (n = 20). Source data for **d** are provided as a Source Data file.

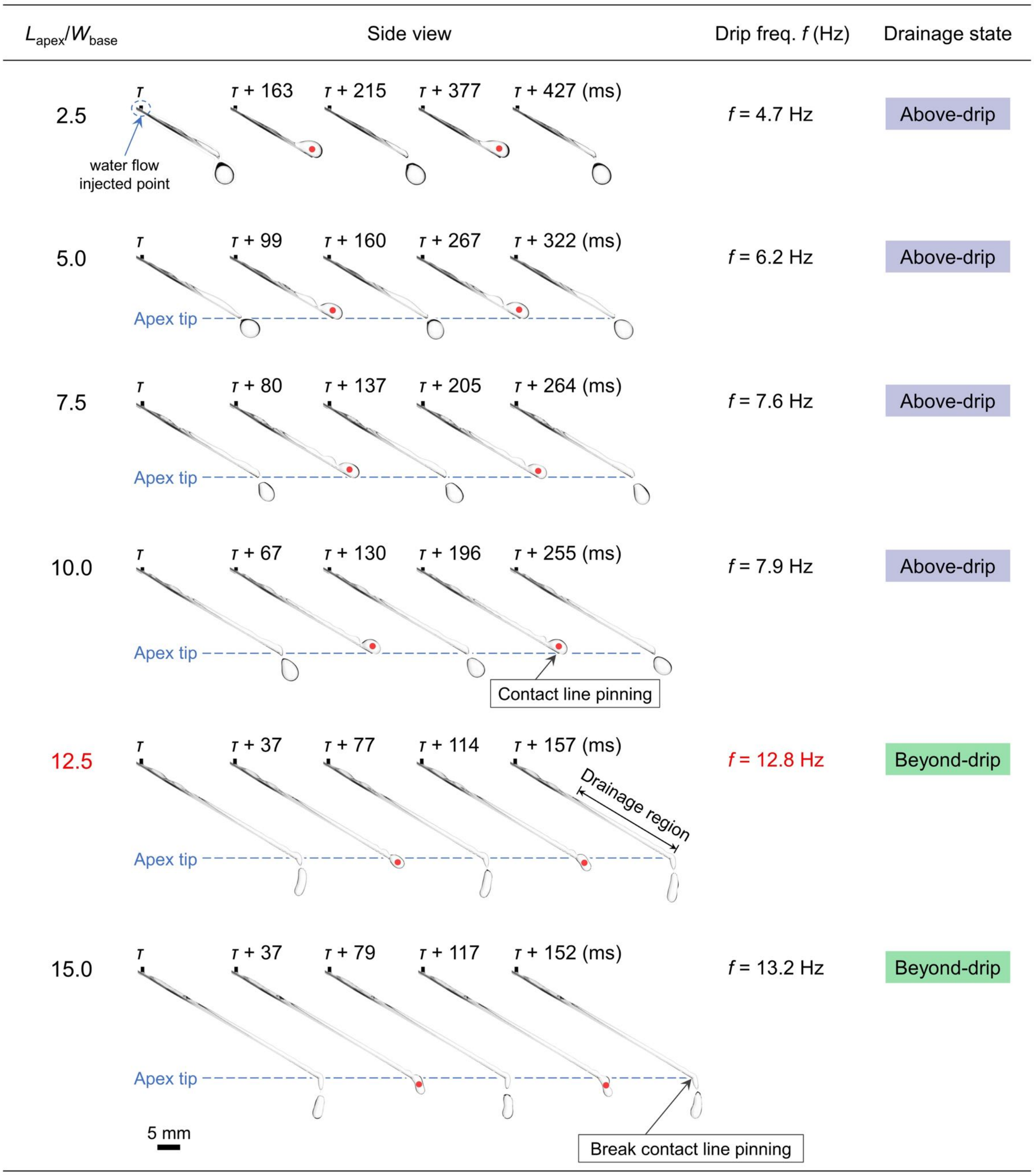

Supplementary Figure 7 | Side view of water flow dynamics on PET-based artificial bodhi leaf apices. L_{apex}/W_{base} ranged from 2.5 to 15.0. Three continuous droplets were shown for each apex sample. Q = 12.0 mL min^{-1} and β = 30°.

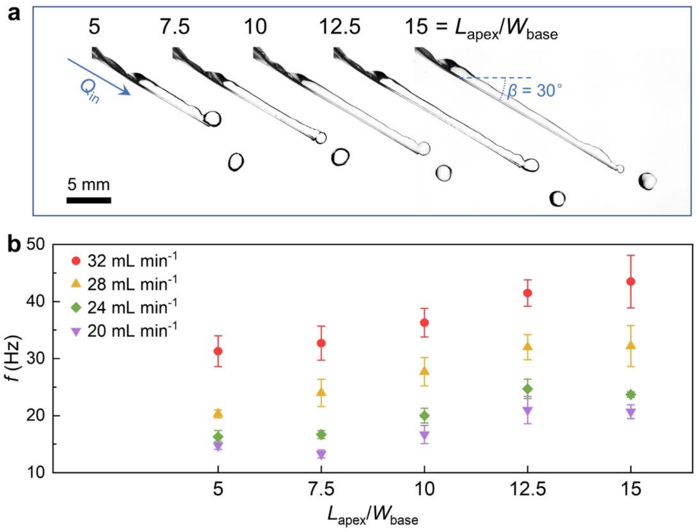

Supplementary Figure 8 | Effects of long-tail L_{apex}/W_{base} on water drainage frequency f under three-needle experimental setup. a Drainage dynamics of artificial bodhi leaf apex with different L_{apex}/W_{base} values. Q_{in} = 32.0 mL min^{-1} and β = 30°. **b** Variation of water drainage frequency f with different L_{apex}/W_{base} values. the drainage frequency f increased with L_{apex}/W_{base} and reached a plateau value after L_{apex}/W_{base} reached 12.5. Data in **b** is shown as mean ± SD, and the error bar represents SD (n = 3 independent experiments). Source data for **b** are provided as a Source Data file.

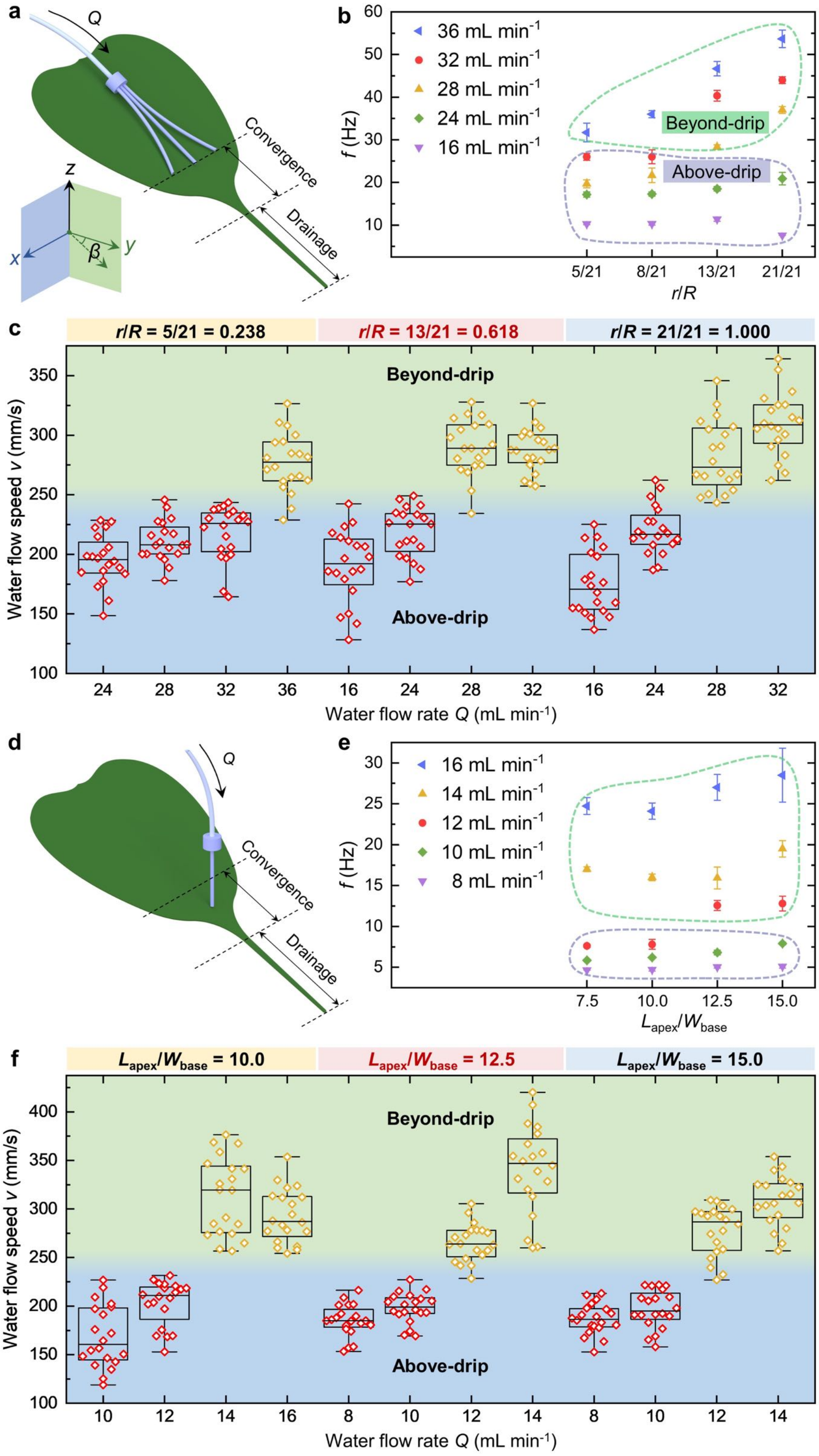

Supplementary Figure 9 | Effects of reverse curvature r/R and long-tail L_{apex}/W_{base} on water flow speed. a Scheme of the three-needle experimental setup for the evaluation of different reverse curvature r/R. **b** Water drainage frequency f of various r/R values at flow rate Q of 16.0 ~ 36.0 mL min^{-1} and β = 30°. Data extracted from Fig. 2e. The light-blue and mint-green dashed curves denote Above-drip and Beyond-drip states, respectively. **c** Measured water flow speed v of three r/R values at 16.0 ~ 36.0 mL min^{-1} and β = 30°. The measured v in the Beyond-drip state was higher than that in the Above-drip state, and the critical speed v_c was roughly located in 230 - 260 mm/s. Note that tested Q values for each sample might be different,

to find out the range of critical speed v_c value. **d** Scheme of single-needle experimental setup for the evaluation of different long-tail L_{apex}/W_{base}. **e** Water drainage frequency f of various L_{apex}/W_{base} values at 8.0 ~ 16.0 mL min^{-1} and β = 30°. Data extracted from Fig. 2h. **f** Measured v of three L_{apex}/W_{base} values at Q of 8.0 ~ 16.0 mL min^{-1} and β = 30°. The measured v in the Beyond-drip state was higher than that in the Above-drip state, and the critical speed v_c was roughly located in 230 - 260 mm/s. Data in (**b** and **e**) are shown as mean ± SD, and the error bar represents SD (n = 3 independent experiments). For the box plots in (**c** and **f**), the bounds and centre line of boxes show the 25/75 percentiles and median values, and the upper and lower whiskers show maxima and minima values (n = 20). Source data for (**b**, **c**, **e and f**) are provided as a Source Data file.

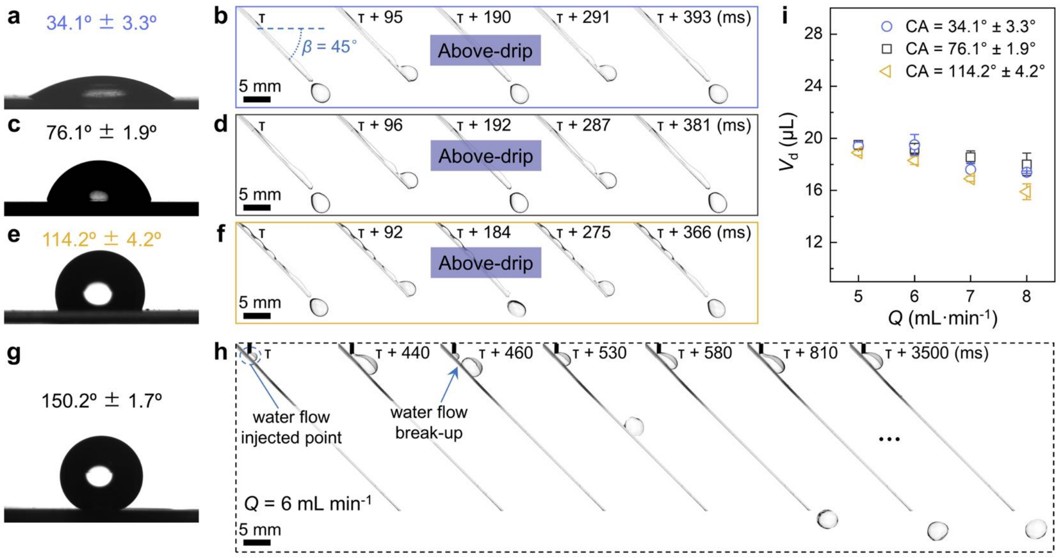

Supplementary Figure 10 | Effects of wettability on drainage behavior of PET-based bodhi leaf apex. Water contact angle and corresponding drainage dynamics on hydrophilic (**a**, **b**), untreated (**c**, **d**), hydrophobic (**e**, **f**), and superhydrophobic (**g**, **h**) PET-based leaf apices. **i** Variation of drip volume V_d with injection flow rate Q at β of 45° under three wettability states. Taking β = 45° as an example. In the case of hydrophilic, untreated, and hydrophobic PET substrates, the injected water formed a continuous liquid stream on PET-based leaf apices and exhibited Above-drip state at Q of 5.0 ~ 8.0 mL min^{-1}. The drip volume V_d was stable and generally close. When it came to superhydrophobic PET-based leaf apex, the injected water flow broke up and formed droplets near the water flow injected point, leading to a discontinuous and unstable drainage process. Therefore, our artificial bodhi leaf apex performed drainage behaviors well under a suitable CA range of 30° ~ 110°. Data in (**i**) is shown as mean ± SD, and the error bar represents SD (n = 3 independent experiments). Source data for (**i**) are provided as a Source Data file.

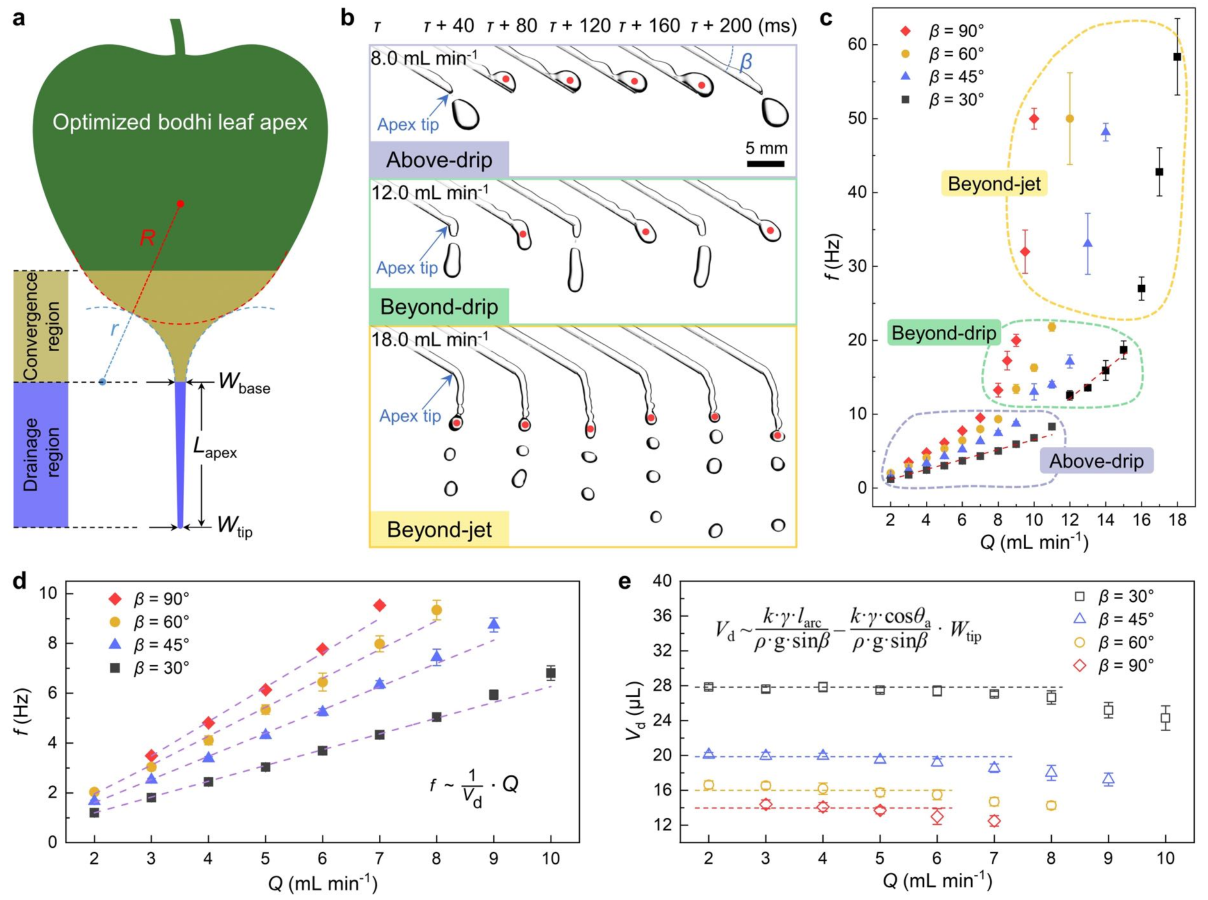

Supplementary Figure 11 | Three drainage states of water flow on the PET-based optimized bodhi leaf apex. a The optimized bodhi leaf apex with r/R = 0.618, L_{apex}/W_{base} = 12.5 and W_{tip} = 1.0 mm. **b** Drainage dynamics at the flow rate Q of 8.0, 12.0, 18.0 mL min^{-1} with the inclination angle β of 30°. Time sequence images in side view every 40.0 ms. **c** Variation of drip frequency f with flow rate Q at β from 30° to 90°. The dashed curves denoted the Above-drip, Beyond-drip, and Beyond-jet states termed in (**b**). The slope of $f \sim Q$ in the Beyond-drip state increased compared to that of the Above-drip state, indicating a smaller drip volume V_d (on average) in the Beyond-drip state. **d** The drip frequency f scaled with Q in the Above-drip state. R^2 = 0.99 for the linear fittings. **e** Variation of measured drip volume V_d with flow rate Q in the Above-drip state. At β = 30°, V_d kept constant at Q of 2.0 - 8.0 mL min^{-1}. In the Above-drip state, the theoretical V_d values (indicated by dashed lines) decreased when β increased from 30° to 90°. The coefficient factor k was calculated to be 0.93 based on the measured V_d values at β of 30° - 90° (assuming l_{arc} = W_{tip} = 1.0 mm, θ_a = 180°). Data in (**c**, **d** and **e**) is shown as mean ± SD, and the error bar represents SD (n = 3 independent experiments). Source data for (**c**, **d** and **e**) are provided as a Source Data file.

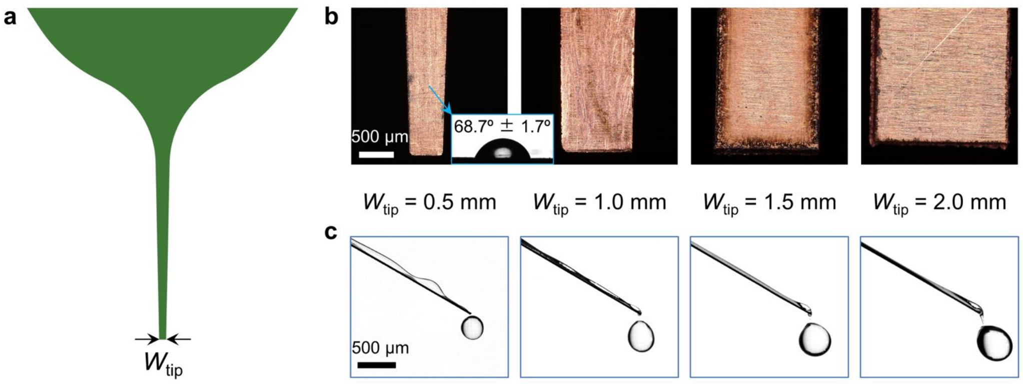

Supplementary Figure 12 | Experimental design of W_{tip}. a Model of Cu-based bodhi leaf apex. **b** Light microscopy of Cu-based bodhi leaf apices with W_{tip} of 0.5, 1.0, 1.5, 2.0 mm. Inset shows water CA of Cu-based leaf apex. **c** Side view of drip volume V_d on bodhi leaf apex with different W_{tip} at Q of 4.0 mL min^{-1} and β of 30°.

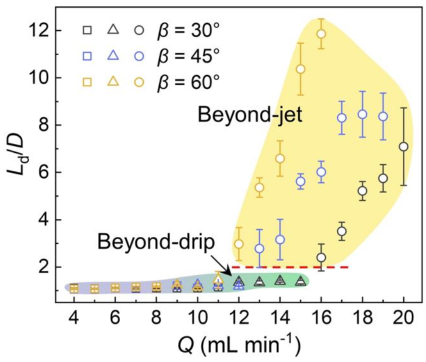

Supplementary Figure 13 | Definition of transition from Beyond-drip state to Beyond-jet state based on the ratio L_d/D. The red dashed line indicated $L_d/D = 2.0$. Data is shown as mean ± SD, and the error bar represents SD (n = 3 independent experiments). Source data are provided as a Source Data file.

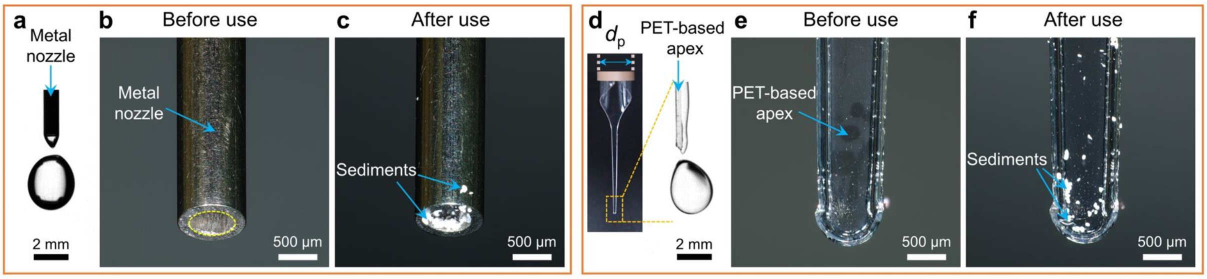

Supplementary Figure 14 | Comparison of the commercial metal nozzle and PET-based BLAM emitter. a Water droplet dripping off a metal nozzle (inner diameter of 600 μm, outer diameter of 900 μm). Optical image of the metal nozzle before use (**b**) and after sequentially draining 100 mL nylon-particle-contained water (**c**). Nylon particles served as probable sediments in the water. **d** Water droplet dripping off the PET-based BLAM emitter. Optical image of PET-based BLAM emitter before use (**e**) and after sequentially draining 100 mL nylon-particle-contained water (**f**). For metal nozzle emitter, the nozzle diameter was small. The sediments (nylon particles here) tended to aggregate at the nozzle outlet. The sediment blockage would decrease the nozzle's inner diameter, increase the flow resistance, and even completely block the nozzle outlet if without cleaning. For the BLAM emitter, water first flowed off a big nozzle (inner diameter d_p = 8 mm, see Supplementary Figure 15 for detailed parameters) along the long tail of the BLAM emitter, and then dripped a small droplet controlled by the W_{tip} of the BLAM emitter. Although sediments could deposit on the BLAM emitter, the combination of a “big nozzle” and a “small open emitter” reduced the risk of nozzle blockage.

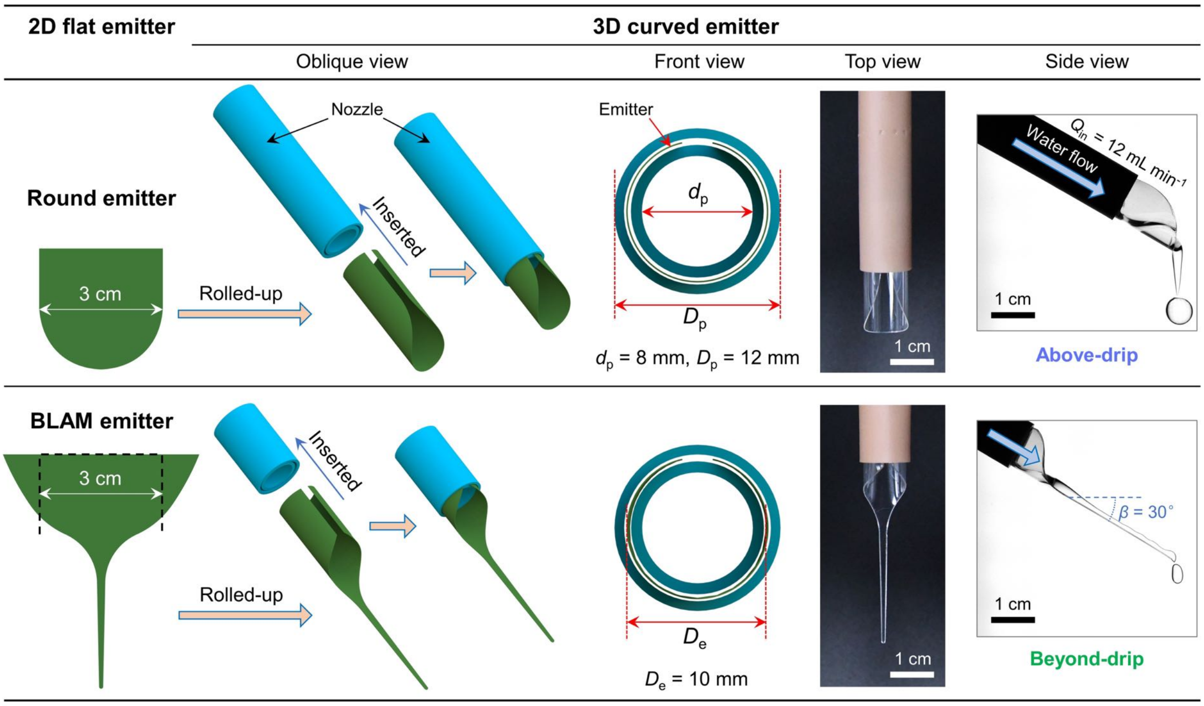

Supplementary Figure 15 | Sketch of the manufacturing process of curved emitters. The 2D round emitter contained a semicircle with a diameter of 3 cm. The 2D BLAM emitter was cut along the black dashed lines from the optimized artificial bodhi leaf with r/R = 0.618 and L_{apex}/W_{base} = 12.5. The round and BLAM emitters used in irrigation applications are all PET film. Note that the front view sketches are specifically enlarged to clearly show the structure parameters.

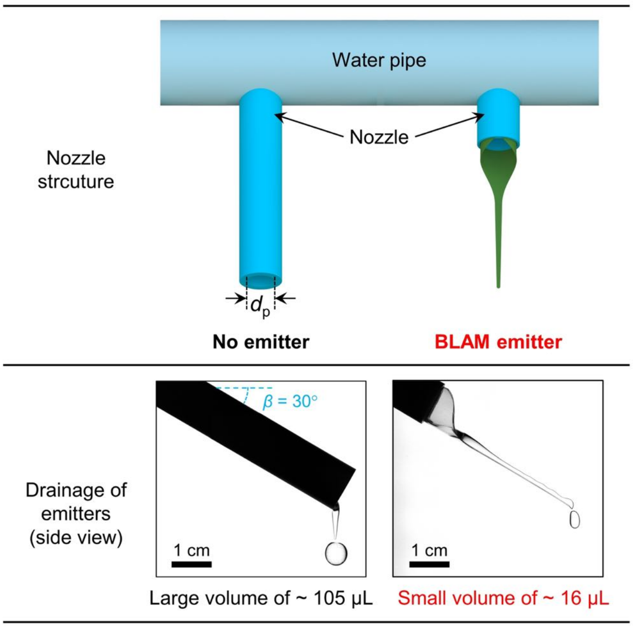

Supplementary Figure 16 | Reduced drip volume by the curved BLAM emitter. The nozzle with inner diameter d_p of 8.0 mm emitted large water droplets with V_d of ≈ 105 μL at β = 30°. The introduction of a curved BLAM emitter could significantly reduce V_d to ≈ 16 μL.

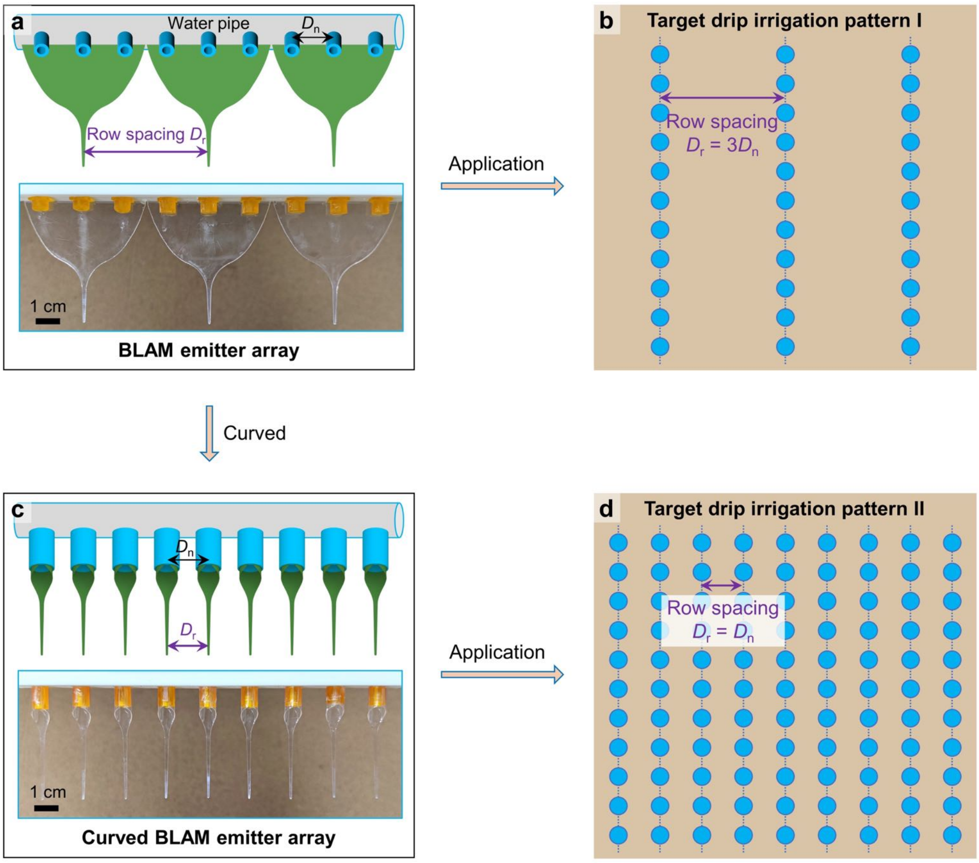

Supplementary Figure 17 | BLAM emitter arrays for drip irrigation application. a Sketch and optical photo of 2D flat BLAM-emitter array. Suppose that there was a water pipe with nine fixed nozzles. The BLAM emitter could converge three water streams from three neighboring nozzles into one merged water flow. Thus, the mobile 2D flat BLAM-emitter array could yield three droplet lines with large row spacing as target drip irrigation pattern I in (**b**). The row spacing of drip irrigation, D_r, equaled $3D_n$, where D_n was the distance between two neighboring nozzles. **c** Sketch and optical photo of curved BLAM-emitter array. A 2D flat BLAM-emitter could be curved and inserted into the nozzle. The mobile curved BLAM-emitter array could yield nine droplet lines with small row spacing as target drip irrigation pattern II in (**d**), where the row spacing D_r equaled D_n. Therefore, we can achieve precise and controllable drip irrigation with adjustable row spacing by switching between flat BLAM emitter array and curved BLAM emitter array, or regulating the number of water streams (three streams here) for convergence.

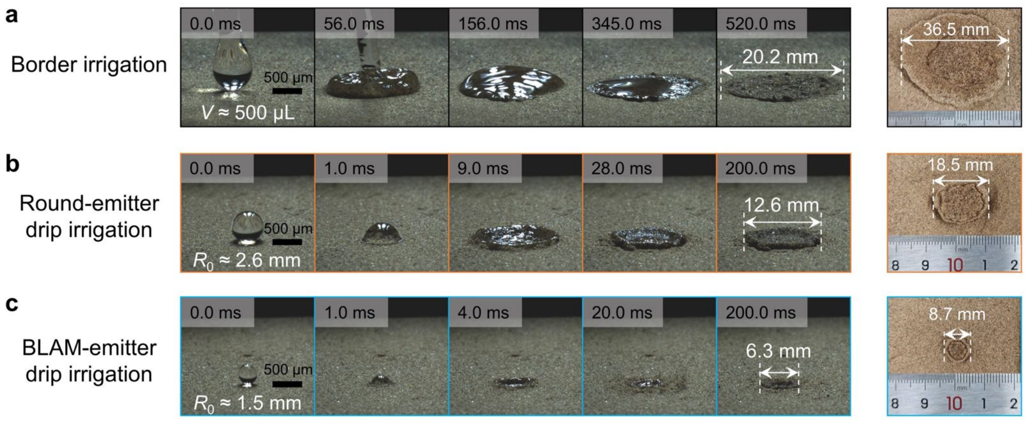

Supplementary Figure 18 | Impacting and spreading dynamics of water droplets on sandy soil under three irrigation modes. a In border irrigation case, water (≈ 500 μL) was directly poured onto the sandy soil surface. For the round-emitter drip irrigation (**b**) and BLAM-emitter drip irrigation (**c**), the water droplets fell freely from 20.0 cm above the sandy soil surface. The impact velocity was 2.0 m/s. The diameter of the final sand block was larger than that of the wetted area after the retracting is finished.

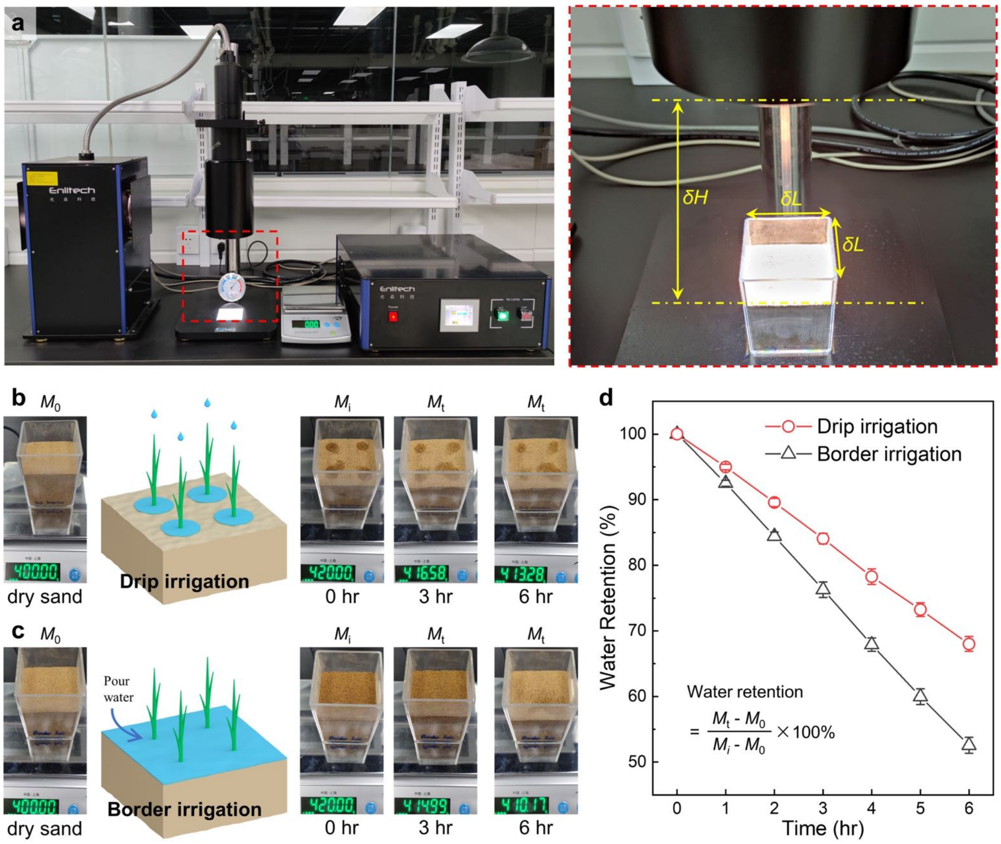

Supplementary Figure 19 | Water evaporation rates under Drip irrigation and Border irrigation. a Experimental setup. A solar simulator was used, with light power of 1,000 W/m^2 and an illuminated area 7 × 7 cm^2, δH = 18 cm, δL = 7 cm. **b** In the drip irrigation model, 20 g water was dripped dropwise onto four crop sites. **c** In the border irrigation model, 20 g of water was uniformly poured onto the sandy soil surface. **d** Water retention percent of drip irrigation and border irrigation models after being continuously exposed to the solar simulator in 6 hours. Water retention is calculated as $(M_t - M_0) / (M_i - M_0) \times 100\%$, where M_0 was the weight of the initial dry sand (400.00 g), M_i was the weight of sand immediately after irrigated with about 20 mL water (420.00 g), and M_t was the weight of irrigated sand after exposed with time t (hour), respectively. Three repeated experiments were performed for each irrigation model, with arithmetic mean value and standard deviation drawn in the graph. The water retention of drip irrigation was higher compared to that of border irrigation in 6 hours, indicating reduced soil evaporation in the drip irrigation case. Data in (**d**) is shown as mean ± SD, and the error bar represents SD (n = 3 independent experiments). Source data for (**d**) are provided as a Source Data file.

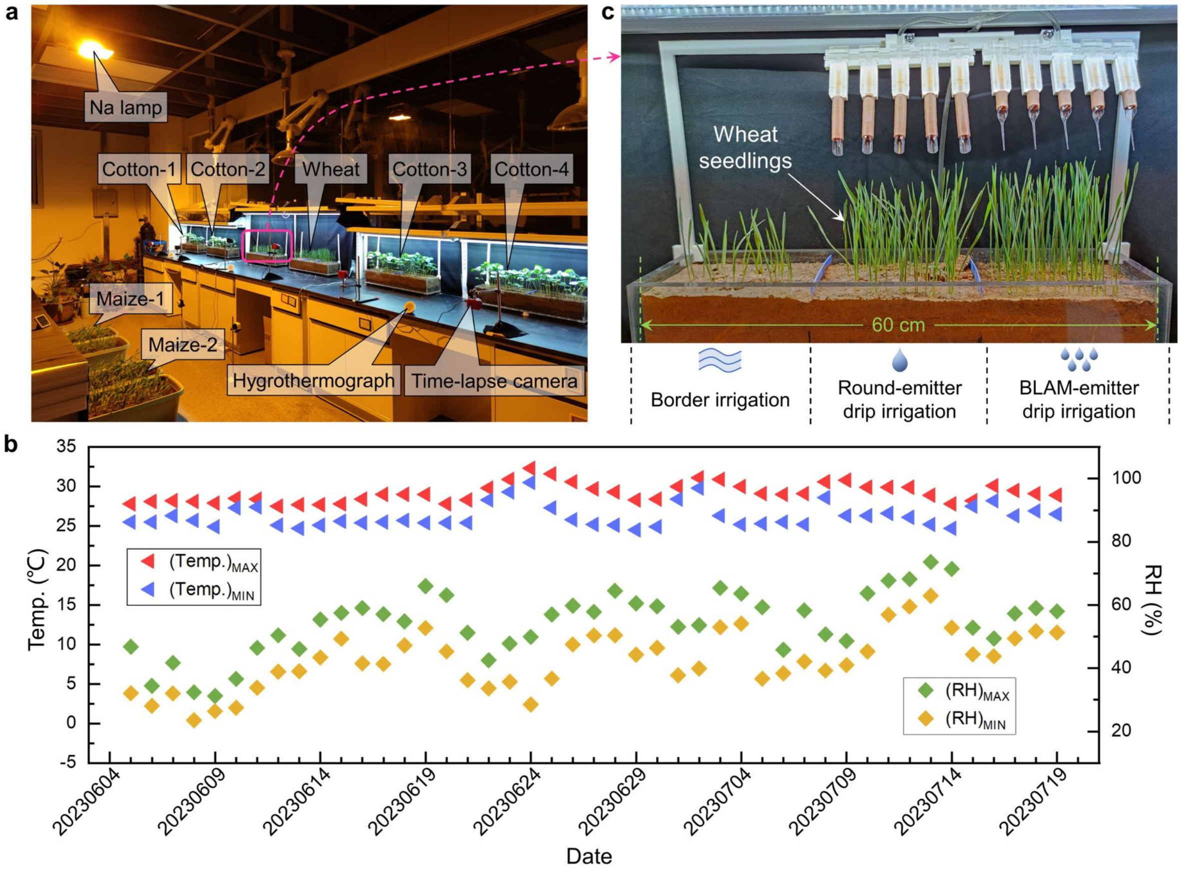

Supplementary Figure 20 | Indoor crop seedling growth. a The indoor laboratory environment for crop seedling growth experiments. For indoor crop seedling growth, the simulated light source was provided to the crop seedlings for 12 hours daily (from 9:00 to 21:00). **b** The daily variation of the maximum (minimum) temperature and relative humidity (RH) of the indoor laboratory environment, which were recorded daily by a hygrothermograph during the crop seedling growth experiments (June & July, 2023). **c** The drip irrigation apparatus for wheat seedling growth, showing the curved round-emitter array (left) and curved BLAM-emitter array (right). Source data for (**b**) are provided as a Source Data file.

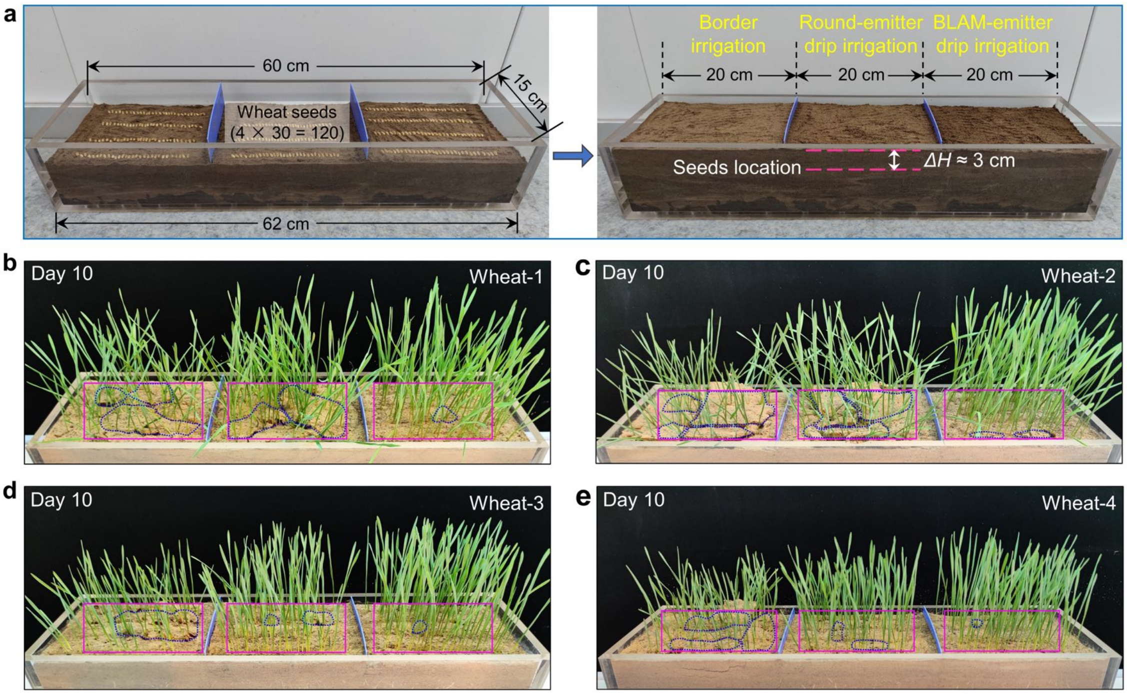

Supplementary Figure 21 | Wheat seedlings grown in acrylic-based planting box. a Wheat seeds pattern before covered by a sandy soil layer with thickness *ΔH* of 3 cm. The inner size of the acrylic-based planting box is 60 × 15 × 10 cm, length × width × depth. The outside length is 62 cm. The sowed area was divided into three zones, with a uniform inner length of 20 cm. **(b, c, d and e)** Four independent replication experiments of wheat seedling growth. The areas marked by purple rectangular boxes were chosen to evaluate the block ratio, where the block area was marked by blue dotted polygons. The detailed statistics results of block ratio were shown in Supplementary Table 1. The time-lapse recording of wheat growth could be found in Supplementary movie 5.

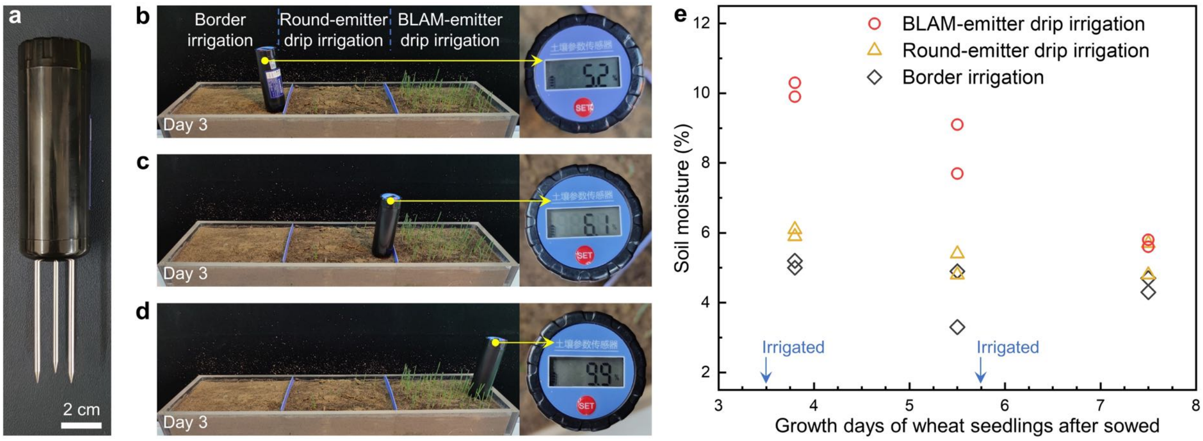

Supplementary Figure 22 | Measurement of soil moisture during the wheat growth process. **a** The used wireless soil parameter sensor, which has three probes (6 cm length) and can measure soil moisture immediately after inserting the probes into the soil. Soil moisture measurement process on soil surface under border irrigation (**b**), round-emitter drip irrigation (**c**), and BLAM-emitter drip irrigation (**d**). **e** Statistics results of soil moisture measurement during the wheat growth process. Two points were measured for each irrigation zone in one measurement process. The measurement was performed every two days. The sandy soil under BLAM-emitter drip irrigation showed higher soil moisture than that of border irrigation and round-emitter drip irrigation. Source data for **e** are provided as a Source Data file.

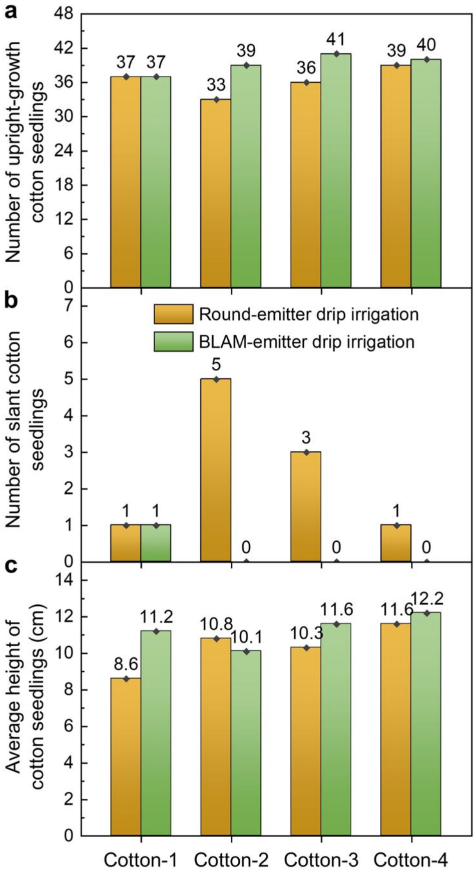

Supplementary Figure 23 | The statistical results of four independent replication experiments of cotton seedling growth. The growing condition of cotton seedlings under two drip irrigation modes in a period of 21 days could be found in Fig. 5e and Supplementary movie 6. Number of upright-growth cotton seedlings (**a**), number of slant cotton seedlings (**b**), and average height of cotton seedlings (**c**) on Day 21 of the cotton seedling growth experiments. The slant cotton seedlings were all labelled with white arrows in Fig. 5e. Source data for (**a**, **b** and **c**) are provided as a Source Data file.

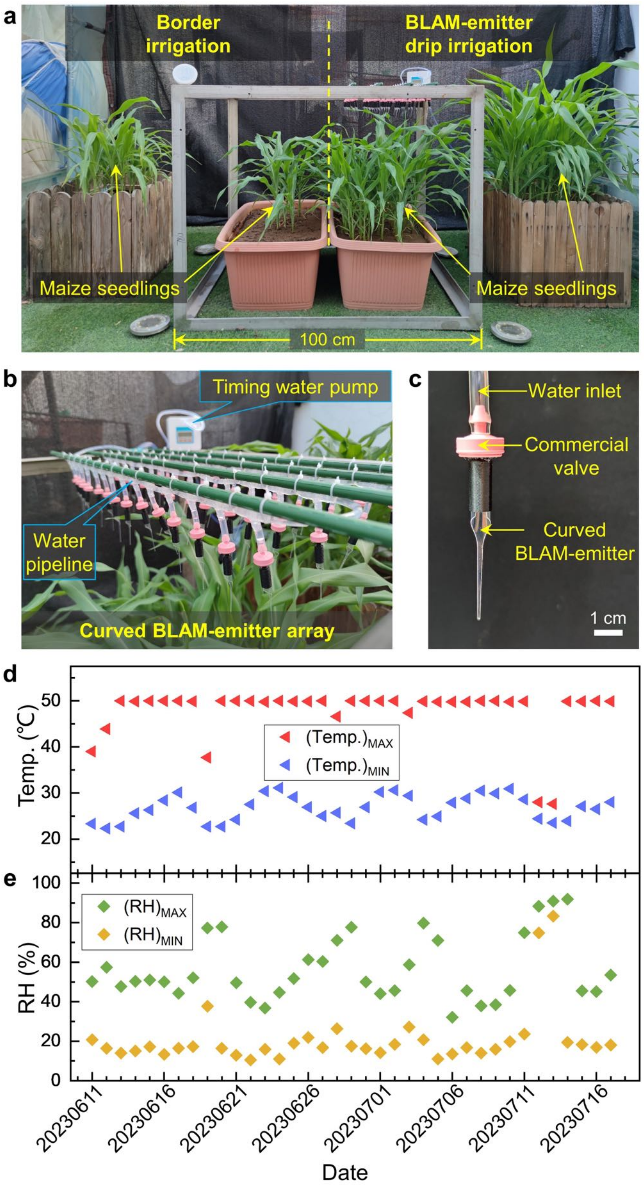

Supplementary Figure 24 | Outdoor maize seedling growth experiments. a Growth condition of maize seedlings under border irrigation and BLAM-emitter drip irrigation after sowed in sandy soil 36 days. **b** The curved BLAM-emitter array used for drip irrigation. **c** The structure of one curved BLAM-emitter. The daily variation of the maximum (minimum) temperature (**d**) and RH (**e**) of the outdoor environment, which were recorded daily by a hygrothermograph during the maize seedling growth experiments (June & July, 2023). Note that during the outdoor maize growth (from June 11, 2023 to July 17, 2023), there have been 30 days with maximum temperature $(Temp.)_{MAX}$ reaching or exceeding 50 °C. Under such hot and drought condition, the maize seedlings under border irrigation grew badly with a poor sprout ratio of 9%, while the maize seedlings under BLAM-emitter drip irrigation grew much better with a sprout ratio of 47% (see Fig. 5g). Such results further confirmed the promoting effect of BLAM-emitter drip irrigation on crop seedling growth. Source data for (**d** and **e**) are provided as a Source Data file.

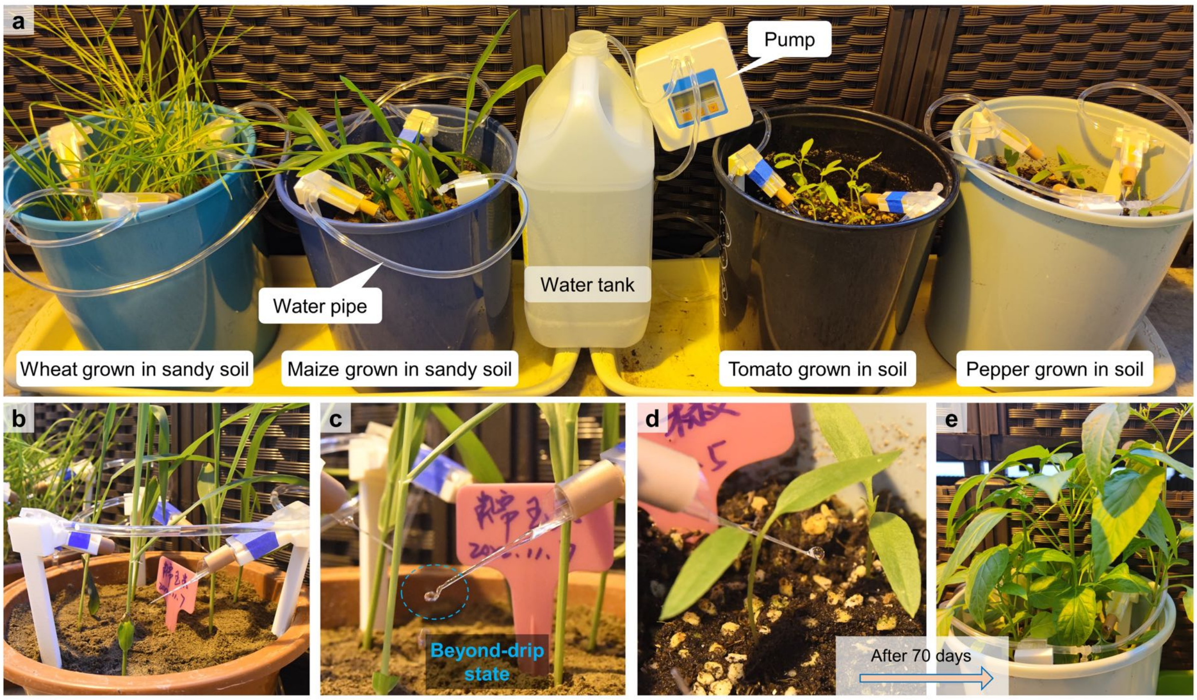

Supplementary Figure 25 | Growth of various crop seedlings under BLAM-emitter drip irrigation. a The self-constructed drip-irrigation system equipped with curved BLAM emitters. A pump connected water tank and BLAM emitters through water pipes. From left to right shown were wheat grown in sandy soil, maize grown in sandy soil, tomato grown in soil, and pepper grown in soil, respectively. **b** BLAM emitters for the drip-irrigation of maize. **c** Drip-irrigation process of the BLAM emitter. The BLAM emitter was mounted at an inclination angle β of 30° and the drainage dynamics belongs to the Beyond-drip state. **d** BLAM emitter for the drip-irrigation of pepper. **e** The pepper seedlings grew well under BLAM-emitter drip irrigation for over two months.

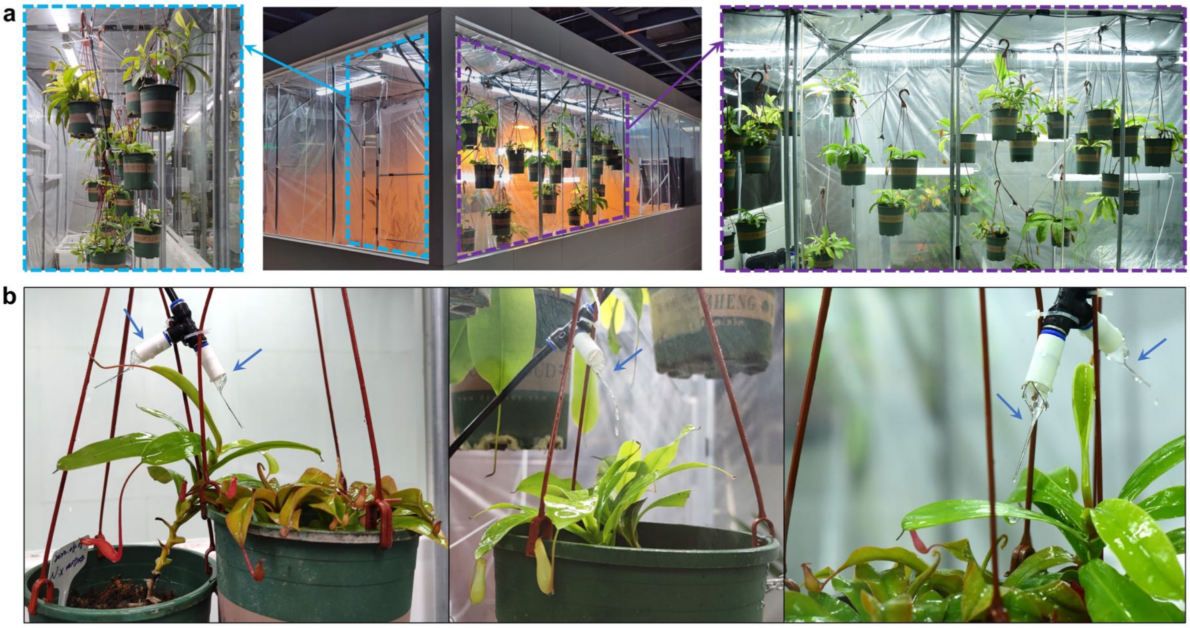

Supplementary Figure 26 | Growth of pitcher plants under BLAM-emitter drip irrigation. **a** Front view, oblique view, and side view of the homemade greenhouse equipped with BLAM-emitter irrigation system. **b** Enlarged images showing the irrigation process of curved BLAM emitters. The blue arrows indicate some of the curved BLAM emitters' location. The pitcher plants grow well under the high-frequency and low-volume dropwise irrigation of BLAM emitters.

Supplementary Table 1. Statistical results of four independent replication experiments of wheat seedling growth under three irrigation modes after sowed for ten days.

Irrigation mode	Border irrigation				Round-emitter drip irrigation				BLAM-emitter drip irrigation			
Experiment number	Wheat -1	Wheat -2	Wheat -3	Wheat -4	Wheat -1	Wheat -2	Wheat -3	Wheat -4	Wheat -1	Wheat -2	Wheat -3	Wheat -4
Total wheat seeds, N_0	120	120	120	120	120	120	120	120	120	120	120	120
Numbers of wheat seedlings on the tenth day, N_{10}	71	88	80	91	90	93	93	102	95	110	107	108
Sprout ratio, N_{10}/N_0	59.2%	73.3%	66.7%	75.8%	75.0%	77.5%	77.5%	85.0%	79.2%	91.7%	89.2%	90.0%
Numbers of slant-grown wheat seedlings, N_s	17	36	7	22	14	32	2	4	5	8	1	1
Slant ratio, N_s/N_{10}	23.9%	40.9%	8.8%	24.2%	15.6%	34.4%	2.2%	3.9%	5.3%	7.3%	0.9%	0.9%
Measured area, S_0 (cm^2) (purple rectangular boxes in Fig. 5a bottom, and Supplementary Figure 21 b-e)	202.4	220.9	199.4	211.6	202.4	220.9	199.4	211.6	202.4	220.9	199.4	211.6
Block area, S_1 (cm^2) (blue dotted polygons in Fig. 5a bottom, and Supplementary Figure 21 b-e)	93.7	142.1	74.6	116.5	101.2	135.5	16.8	24.4	8.1	14.6	5.0	4.0
Block ratio, S_1/S_0	46.3%	64.3%	37.4%	55.1%	50.0%	61.3%	8.4%	11.5%	4.0%	6.6%	2.5%	1.9%
