# Supplementary material for: Efficient agricultural drip irrigation inspired by fig leaf morphology
Source: Nat Commun. 2023 Sep 23;14:5934. doi: 10.1038/s41467-023-41673-0 (PMC10518012; doi:10.1038/s41467-023-41673-0)
Supplement: Supplementary file 2 — Reporting Summary [file 41467_2023_41673_MOESM2_ESM.pdf]

nature portfolio

Corresponding author(s): Zhichao Dong
Last updated by author(s): Sep 11, 2023

Reporting Summary

Nature Portfolio wishes to improve the reproducibility of the work that we publish. This form provides structure for consistency and transparency in reporting. For further information on Nature Portfolio policies, see our Editorial Policies and the Editorial Policy Checklist.

Statistics

For all statistical analyses, confirm that the following items are present in the figure legend, table legend, main text, or Methods section.

n/a	Confirmed	
[ ]	[x]	The exact sample size (*n*) for each experimental group/condition, given as a discrete number and unit of measurement
[x]	[ ]	A statement on whether measurements were taken from distinct samples or whether the same sample was measured repeatedly
[x]	[ ]	The statistical test(s) used AND whether they are one- or two-sided<br>*Only common tests should be described solely by name; describe more complex techniques in the Methods section.*
[x]	[ ]	A description of all covariates tested
[x]	[ ]	A description of any assumptions or corrections, such as tests of normality and adjustment for multiple comparisons
[ ]	[x]	A full description of the statistical parameters including central tendency (e.g. means) or other basic estimates (e.g. regression coefficient) AND variation (e.g. standard deviation) or associated estimates of uncertainty (e.g. confidence intervals)
[x]	[ ]	For null hypothesis testing, the test statistic (e.g. *F*, *t*, *r*) with confidence intervals, effect sizes, degrees of freedom and *P* value noted<br>*Give P values as exact values whenever suitable.*
[x]	[ ]	For Bayesian analysis, information on the choice of priors and Markov chain Monte Carlo settings
[x]	[ ]	For hierarchical and complex designs, identification of the appropriate level for tests and full reporting of outcomes
[x]	[ ]	Estimates of effect sizes (e.g. Cohen's *d*, Pearson's *r*), indicating how they were calculated

Our web collection on statistics for biologists contains articles on many of the points above.

Software and code

Policy information about availability of computer code

Data collection	Photron Fastcam Viewer 4
Data analysis	Photron Fastcam Viewer 4, Microsoft Excel 365, Origin 2021

For manuscripts utilizing custom algorithms or software that are central to the research but not yet described in published literature, software must be made available to editors and reviewers. We strongly encourage code deposition in a community repository (e.g. GitHub). See the Nature Portfolio guidelines for submitting code & software for further information.

Data

Policy information about availability of data

All manuscripts must include a data availability statement. This statement should provide the following information, where applicable:

- Accession codes, unique identifiers, or web links for publicly available datasets
- A description of any restrictions on data availability
- For clinical datasets or third party data, please ensure that the statement adheres to our policy

All data needed to evaluate the conclusions in the paper are available in the main text or the supplementary materials. Source data are provided in this paper.

Human research participants

Policy information about studies involving human research participants and Sex and Gender in Research.

Reporting on sex and gender	N/A
Population characteristics	N/A
Recruitment	N/A
Ethics oversight	N/A

Note that full information on the approval of the study protocol must also be provided in the manuscript.

Field-specific reporting

Please select the one below that is the best fit for your research. If you are not sure, read the appropriate sections before making your selection.

☐ Life sciences ☐ Behavioural & social sciences ☒ Ecological, evolutionary & environmental sciences

For a reference copy of the document with all sections, see nature.com/documents/nr-reporting-summary-flat.pdf

Ecological, evolutionary & environmental sciences study design

All studies must disclose on these points even when the disclosure is negative.

Study description	In this study, the effects of different irrigation modes on crop growth were explored. Three irrigation modes were discussed, i.e., traditionally used border irrigation, round-emitter drip irrigation, and the biomimetic BLAM-emitter drip irrigation with high drip frequency and small drip volume characteristics. The tested crops included wheat, cotton, and maize. Three evaluation indexes were founded and examined, i.e., soil block ratio, crop sprout ratio, and slant-growth ratio.
Research sample	The tested crops include wheat, cotton, and maize. For wheat cultivation, 100 seeds were randomly chosen and sown in sandy soil under each irrigation mode. For cotton, 48 seeds were randomly chosen and sown in sandy soil under each irrigation mode. For maize, 100 seeds were randomly chosen and sown in soil under border irrigation and BLAM-emitter drip irrigation. All the seeds were bought and used as received without pretreatments before cultivation.
Sampling strategy	N/A
Data collection	S. L. recorded the crop growth and collected the images and time-lapse videos for wheat, cotton, and maize.
Timing and spatial scale	The wheat and cotton growth was performed in a cultivation box (inner size 60 × 15 × 10 cm, length × width × depth). The time periods in the cultivation of wheat, cotton, and maize were 10 days, 21 days, and 36 days, respectively.
Data exclusions	N/A
Reproducibility	Four groups of indoor wheat cultivations were performed. Four groups of indoor cotton cultivations were performed simultaneously. Three groups of maize cultivations were performed, both indoors and outdoors. All attempts to repeat the experiments were successful.
Randomization	Randomization was non-applicable in our study, as all the seedlings were recorded and counted up in each crop cultivation experiment.
Blinding	N/A

Did the study involve field work? ☒ Yes ☐ No

Field work, collection and transport

Field conditions	During the outdoor maize cultivation, the temperature and relative humidity (RH) were recorded daily by a hygrothermograph during the maize cultivation.
Location	The outdoor maize cultivation was performed in June & July, 2023 in Beijing (40°N, 116°E), China.
Access & import/export	N/A

Disturbance	N/A

Reporting for specific materials, systems and methods

We require information from authors about some types of materials, experimental systems and methods used in many studies. Here, indicate whether each material, system or method listed is relevant to your study. If you are not sure if a list item applies to your research, read the appropriate section before selecting a response.

Materials & experimental systems

n/a	Involved in the study	
☒	☐	Antibodies
☒	☐	Eukaryotic cell lines
☒	☐	Palaeontology and archaeology
☒	☐	Animals and other organisms
☒	☐	Clinical data
☒	☐	Dual use research of concern

Methods

n/a	Involved in the study	
☒	☐	ChIP-seq
☒	☐	Flow cytometry
☒	☐	MRI-based neuroimaging
