# Supplementary material for: Efficient agricultural drip irrigation inspired by fig leaf morphology
Source: Nat Commun. 2023 Sep 23;14:5934. doi: 10.1038/s41467-023-41673-0 (PMC10518012; doi:10.1038/s41467-023-41673-0)
Supplement: Supplementary file 3 — Description of Additional Supplementary Files [file 41467_2023_41673_MOESM3_ESM.pdf]

Description of Additional Supplementary Files

File name: Supplementary Movie 1.

Description: Reverse curvature reshaped flow hydrodynamics and drop separation centroid.

File name: Supplementary Movie 2.

Description: Long tail enhanced water drip frequency *f*.

File name: Supplementary Movie 3.

Description: Three drainage behaviors, i.e., Above-drip state, Beyond-drip state, and Beyond-jet state.

File name: Supplementary Movie 4.

Description: The mobile drip irrigation prototype.

File name: Supplementary Movie 5.

Description: Indoor wheat growth under border irrigation, round-emitter drip irrigation, and BLAM-emitter drip irrigation.

File name: Supplementary Movie 6.

Description: Indoor cotton growth under round-emitter drip irrigation and BLAM-emitter drip irrigation.

File name: Supplementary Movie 7.

Description: Indoor maize growth under border irrigation and BLAM-emitter drip irrigation.

File name: Supplementary Movie 8.

Description: Outdoor maize growth under border irrigation and BLAM-emitter drip irrigation.
